# Trends in socioeconomic inequality in e-cigarette use among adolescents in South Korea

**DOI:** 10.18332/tid/194099

**Published:** 2024-10-17

**Authors:** Gaoran Chen, Hong Lu, Wenqi Chen, Shaojie Qi, Wenbin Du

**Affiliations:** 1 Research Institute of Social Development, Southwestern University of Finance and Economics, Chengdu, China

**Keywords:** socioeconomic inequality, e-cigarette, adolescents, South Korea

## Abstract

**INTRODUCTION:**

The global rise in e-cigarette use among adolescents is alarming, with associated socioeconomic inequalities posing potential public health risks. This study examined trends in the socioeconomic inequality in e-cigarette use among South Korean adolescents to inform future regulatory directions.

**METHODS:**

Socioeconomic inequalities in e-cigarette use among Korean adolescents were assessed using data from the Korea Youth Risk Behavior Web-based Survey (KYRBS) from 2011 to 2023. The Concentration Index, a well-established method for measuring health inequalities, was employed. Additionally, this study investigated how the smoking behaviors of family members and friends influence socioeconomic inequality in e-cigarette use among Korean adolescents, using a decomposition analysis.

**RESULTS:**

The Concentration Index values showed a clear, fluctuating downward trend over 13 years, from -0.12 (95% CI: -0.13 – -0.10) in 2011 to -0.24 (95% CI: -0.26 – -0.21) in 2023. Decomposition analysis revealed that smoking among peer groups, including friends and siblings, was the primary contributor to socioeconomic inequality in e-cigarette use, followed by maternal smoking.

**CONCLUSIONS:**

Socioeconomic inequalities in adolescent e-cigarette use in South Korea are widening, particularly among low socioeconomic status groups. The impact of peer groups on socioeconomic inequalities in e-cigarette use among adolescents is concerning.

## INTRODUCTION

The wide range of flavors, attractive packaging, and limited online marketing regulations for e-cigarettes have rapidly increased their popularity among adolescents^1^. According to WHO reports, the prevalence of e-cigarette usage among those aged 13–15 years exceeds that among adults in all member countries^2^. Nonetheless, using any tobacco product carries risks. Nicotine and other harmful compounds in e-cigarettes may contribute to respiratory and cardiovascular diseases^3^. E-cigarettes may also have adverse effects on the neurodevelopment of adolescents^4^. Research has also shown that e-cigarettes can negatively impact the mental well-being of teenagers^5^. Furthermore, adolescents who use e-cigarettes are more likely to start smoking traditional cigarettes and to use other addictive substances^6^.

Socioeconomic inequalities in adolescent e-cigarette use represent a significant public health issue. Historical evidence in tobacco control suggests that reductions in tobacco product prevalence primarily impact individuals of higher socioeconomic status (SES). Individuals of lower socioeconomic status are the primary consumers of diverse tobacco products^7^. Socioeconomic disparities in adolescent e-cigarette use heighten the susceptibility of youth from lower socioeconomic backgrounds to related illnesses and exacerbate health disparities. Conversely, diseases linked to e-cigarette use can further entrench poverty in populations with low socioeconomic status^8^.

Socioeconomic inequalities in adolescent e-cigarette use stem from multiple factors. Besides increasing tobacco prices, there is limited evidence supporting the effectiveness of current tobacco control measures in reducing tobacco use among individuals of lower socioeconomic status^9^. Instead, individuals from lower socioeconomic backgrounds exhibit reduced responsiveness to current tobacco control policies and measures^10^. Furthermore, the living environment plays a significant role in shaping adolescents’ health behaviors. Parents of adolescents with lower socioeconomic status are more likely to use tobacco products, affecting their parenting style and increasing the likelihood of adolescent exposure to these products^11^. Adolescents of lower socioeconomic status are also more susceptible to peer pressure to initiate tobacco use. Additionally, peer tobacco product use is significantly more prevalent among adolescents from lower socioeconomic backgrounds compared to those from higher socioeconomic backgrounds^12^. These factors combine to draw adolescents from lower socioeconomic backgrounds to tobacco products. It is unclear whether these findings apply to e-cigarette use, highlighting the need for further research and empirical verification.

Since 2007, the Korean government has taken a cautious approach to e-cigarettes, enacting various regulatory measures, particularly to protect youth from the risks associated with these products, including categorizing e-cigarettes as cigarettes and banning their sale to adolescents^13^. However, these measures have not effectively addressed the socioeconomic inequalities associated with e-cigarette use. E-cigarette use is mainly concentrated among adolescents from lower socioeconomic backgrounds^14^. However, socioeconomic status indicators differ significantly between adolescents and adults^15^. Previous research findings may not directly apply to adolescents, and there is limited research on the longitudinal trends of socioeconomic disparities in e-cigarette use in Korea. In addition, research investigating the factors contributing to socioeconomic disparities in adolescent e-cigarette use is lacking. Understanding the long-term trends and underlying factors affecting e-cigarette prevalence and disparities is essential for developing future tobacco control policies. These findings inform the development of strategies to reduce health disparities. Therefore, more discussion is necessary on the socioeconomic inequalities in adolescent e-cigarette use and the factors that influence them.

## METHODS

### Data sources and sample selection

This study is a secondary analysis of the Korea Youth Risk Behavior Web-based Survey (KYRBS), conducted annually by the Department of Disease Management in Korea since 2005. Students aged 12–18 years from 400 middle and 400 high schools participated in this anonymous online survey. The KYRBS has collected data in 19 waves from 2005 to 2023. The KYRBS is representative national data for studying adolescent risk behaviors in Korea. The KYRBS data can be downloaded free of charge from the KYRBS website (http://yhs.cdc.go.kr)^16^.

The KYRBS began investigating issues related to adolescent e-cigarette use in 2011. We used data from KYRBS 2011 to KYRBS 2023, a total of 13 waves, to explore socioeconomic inequality of e-cigarette use among Korean adolescents. The samples were taken from KYRBS 2011 (n=75643), 2012 (n=74186), 2013 (n=72435), 2014 (n=72060), 2015 (n=68043), 2016 (n=65528), 2017 (n=62276), 2018 (n=60040), 2019 (n=57303), 2020 (n=54948), 2021 (n=54848), 2022 (n=51850), and 2023 (n=52880). However, only five of these waves contained the complete study variables needed to decompose socioeconomic inequalities in e-cigarette use. For our decomposition analysis, we selected all five waves that included the variable on the smoking status of adolescents’ friends and family members (KYRBS 2014, 2015, 2016, 2018, and 2021).

### Measurements

*Socioeconomic status*

The measurement of SES has been controversial, particularly for adolescents^17^. In previous studies, parental or family SES has usually been used as a proxy of SES^18^ or to assess adolescents’ subjective SES^19^. However, adolescents’ SES is influenced by their families’ socioeconomic and school situations^20^. Recent studies highlight academic achievement as an important component of adolescents’ SES, an aspect often overlooked in prior research.

Consequently, this study considers family SES and academic performance in measuring the SES of adolescents. Parents’ education measured adolescents’ SES, subjective family economic status, and academic performance. Subjective family economic status was used as a proxy for household income^19^ because it is difficult for adolescents to report their households’ specific income accurately. Subjective family economic status was assessed on a 5-point scale from ‘very poor’ to ‘very rich’. Parents’ education level were categorized as junior high school, high school, and university. Adolescents’ academic performance was rated on a 5-point scale from ‘poor’ to ‘excellent’.

Finally, this study employed Principal Component Analysis (PCA), a standard factor analysis method^21^, to integrate parents’ education, subjective family economic status, and adolescents’ academic performance into a single SES index. There are findings supporting the reliability and validity of this method in generating the SES index^8^. The descriptive statistics of this SES index can be found in Supplementary file Table S1.

*Adolescent e-cigarette use*

Adolescent e-cigarette use was assessed with the question: ‘Have you used liquid e-cigarettes with nicotine so far?’. Responses were either ‘yes’ or ‘no’.

*Smoking status of family members*

The smoking status of family members was assessed with the questions: ‘Does your father smoke?’, ‘Does your mother smoke?’, ‘Do your siblings smoke?’ and ‘Do your grandparents smoke?’, with responses ‘yes’ or ‘no’.

*Friend’s smoking status*

Friends’ smoking status was assessed with the question: ‘Do any of your friends smoke?’. Response options were ‘nobody smokes’, ‘a few people smoke’, ‘most of them smoke’, and ‘everyone smokes’. Responses of ‘a few people smoking’, ‘most of them smoke’, and ‘everyone smokes’ were recoded as ‘1’ to indicate having friends who smoke, while ‘nobody smokes’ was recoded as ‘0’ to indicate no smoking friends.

### Statistical analysis

Initially, a descriptive analysis was conducted to summarize the variables. Subsequently, we used crude e-cigarette prevalence rates (CPR), calculated as the number of adolescents using e-cigarettes divided by a total number of youths surveyed, to measure e-cigarette use among adolescents across different years.

The Concentration Index is a useful tool for measuring health inequalities, helping us understand the distribution of health conditions (e.g. health service use or health outcomes) across groups of different socioeconomic status, and is widely used in the fields of health economics and public health^22^. We estimated the inequality in e-cigarette use among adolescents by SES using the Erreygers’ method to estimate the Concentration Index^23^. A negative Concentration Index indicates that e-cigarette use is concentrated among lower SES adolescents, while a positive Concentration Index indicates concentration among higher SES adolescents. Additionally, the confidence intervals of the Concentration Index were calculated at the 95% level.

To examine the contribution of the smoking status of adolescents’ friends and family members to the inequality in e-cigarette use among adolescents, a regression-based decomposition analysis was also employed^24^. Adolescent e-cigarette use was first explained using a Generalized Linear Model (family=binomial, link=logit). The absolute contribution of the smoking status of adolescents’ friends and family members could be taken by estimating the explained component. In decomposition, elasticity indicates the percentage change in the Concentration Index resulting from a 1% increase in a given variable while holding other conditions constant. The contribution of each variable to socioeconomic inequality is determined by the product of elasticity and the Concentration Index^25^. The computation and decomposition of the Concentration Index were performed using the R (version 4.3.0) package *Rineq*. A p<0.05 was considered statistically significant.

## RESULTS

Supplementary file Tables S1 and S2 show the results of descriptive statistics. The gender and age distribution of the sample remained stable from 2011 to 2023, with boys comprising approximately 51% and girls approximately 49%, maintaining an average age of around 15 years. According to adolescents’ subjective family economic situation, most rated their family’s economic situation as medium. The percentage of adolescents who considered their family’s economic situation very poor decreased, while those rating it as very rich increased. Over the 13 years, there was a significant increase in the percentage of adolescents’ parents, particularly mothers, who attained a college education. Despite fluctuations, the majority of adolescents who consider their grades average consistently remained the highest over the 13 years. With regard to smoking among friends and family members of adolescents, smoking rates among fathers, siblings, friends, and grandparents have fluctuated downward, while the percentage of mothers who smoke has increased.

**Figure 1:**
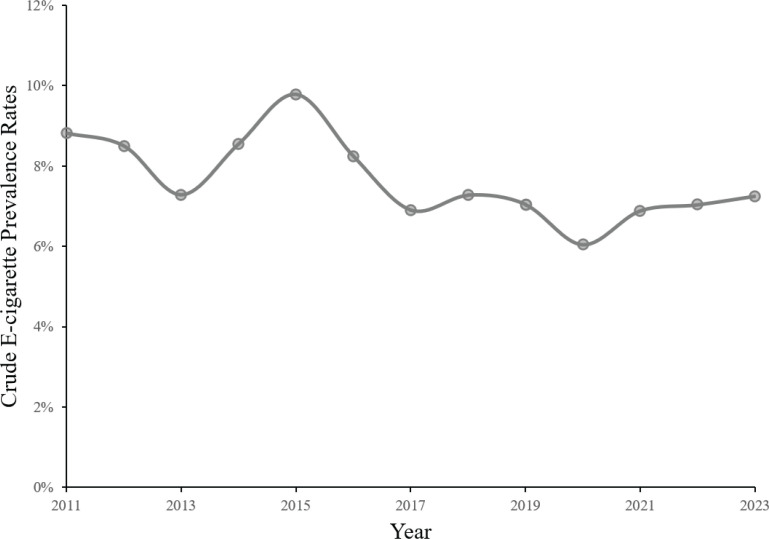
Trends in the crude e-cigarette prevalence rates among adolescents in South Korea, 2011–2023 (N=895278)

Figure 1 shows the crude e-cigarette prevalence rate trends among South Korean adolescents from 2011 to 2023. The crude e-cigarette prevalence rate among Korean adolescents peaked at 9.78% in 2015, followed by a subsequent decline. In 2023, the e-cigarette prevalence rate among Korean adolescents was 7.25%, marking a decrease of 1.57% since 2011.

**Figure 2:**
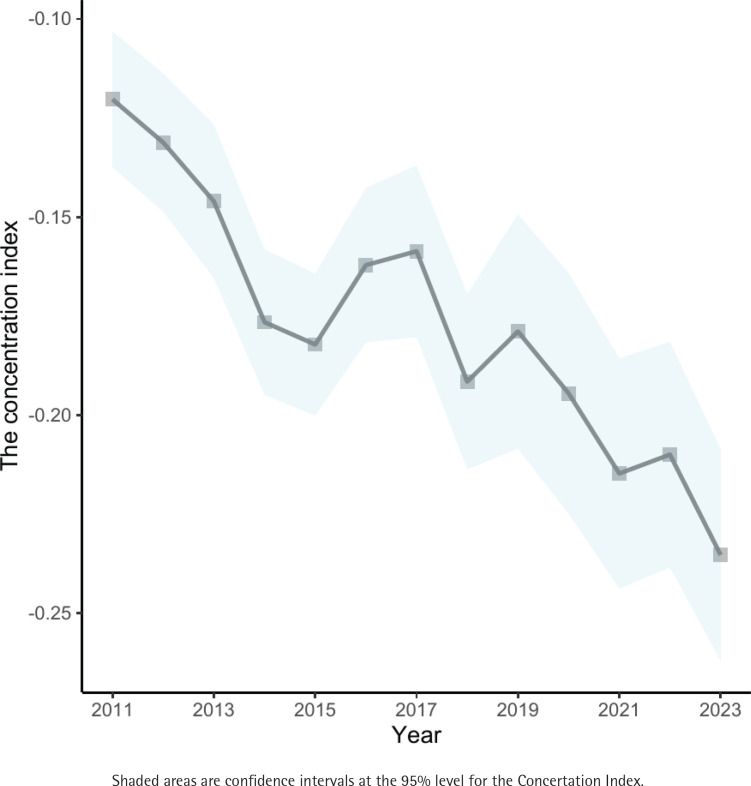
Trends in the Concertation Index among adolescents in South Korea, 2011–2023 (N=895278)

Figure 2 illustrates the trends in the Concentration Index among Korean adolescents from 2011 to 2023. The value of the Concentration Index has been below zero from 2011 to 2023, indicating that e-cigarette use is consistently concentrated among adolescents with low SES. It can be found that the Concentration Index value significantly decreased from -0.12 (95%CI: -0.13 – -0.10) in 2011 to -0.24 (95% CI: -0.26 – -0.21) in 2023. The average annual rate of decline is approximately 0.01. E-cigarette use continues to be increasingly concentrated among Korean adolescents of lower SES.

**Figure 3:**
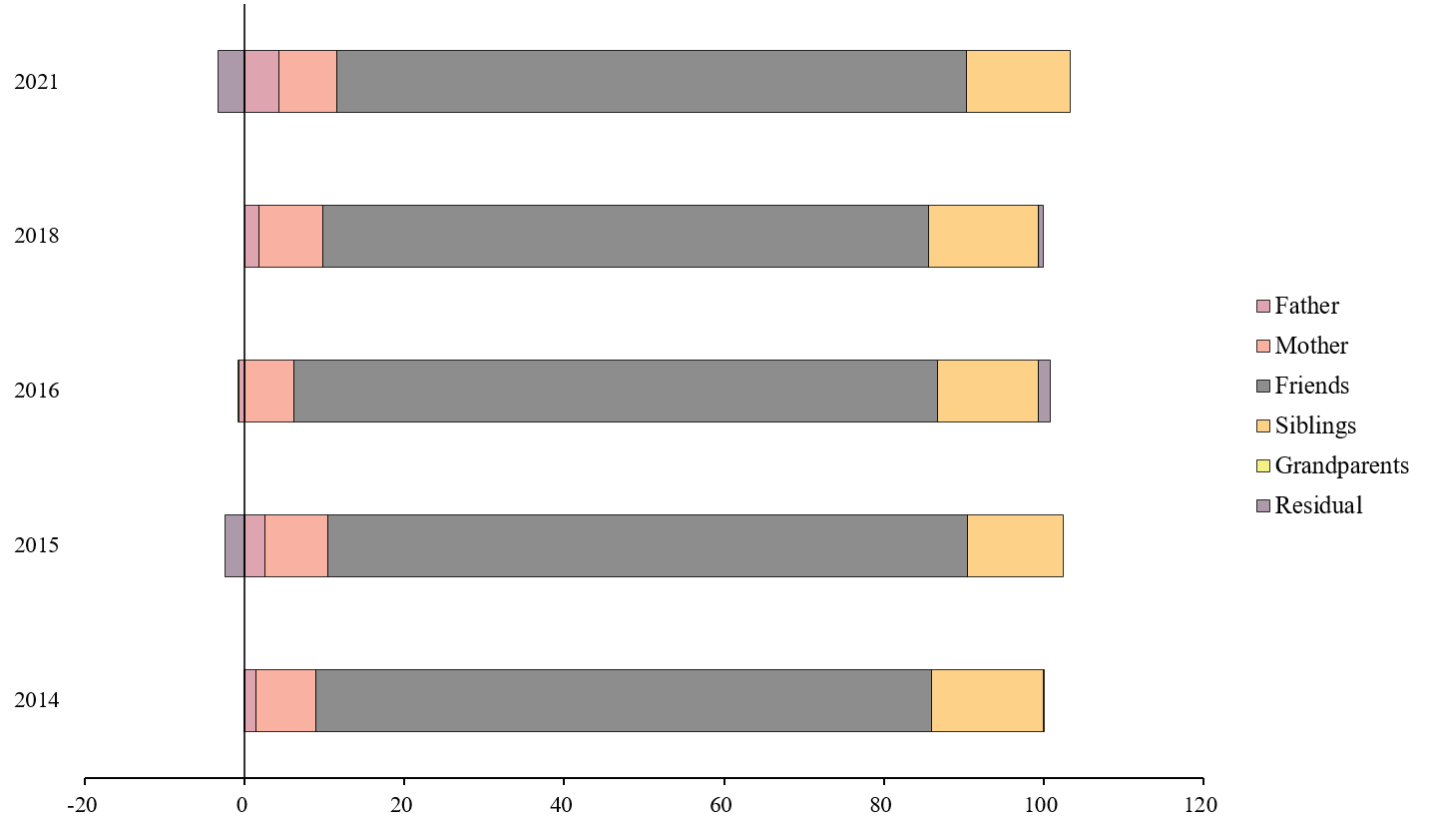
Percentage contribution of each variable to socioeconomic inequality of e-cigarette use among adolescents in South Korea (2014, 2015, 2016, 2018, 2011) (N=317267)

Supplementary file Tables S3–S7 present the decomposition results of the Concentration Index for the years 2014, 2015, 2016, 2018, and 2021. Figure 3 visualizes the main results of the Concentration Index decomposition. The contribution of friends’ and family members’ smoking to socioeconomic inequality in adolescent e-cigarette use was the greatest. In 2021, the contribution of friends’ smoking to socioeconomic inequality in the use of e-cigarettes among adolescents was 78.72%, 7.3% for mothers, and 4.29% for fathers. Besides, the contribution of friends’ and family members’ smoking to socioeconomic inequality in adolescent e-cigarette use remained stable. Across all years, friends’ smoking consistently emerged as the most significant factor contributing to socioeconomic inequalities in e-cigarette use among adolescents, followed by sibling smoking and maternal smoking. The contribution of paternal smoking to socioeconomic inequality in e-cigarette use among adolescents increased from 1.45% in 2014 to 4.29% in 2021. In 2021, contributions to socioeconomic inequality were 78.72% from friends’ smoking, 13.07% from siblings’ smoking, and 7.29% from maternal smoking. Peer groups, including friends and siblings, were the primary contributors to socioeconomic inequality in e-cigarette use among adolescents. In contrast, grandparent smoking status had a minimal impact on this inequality.

## DISCUSSION

Prior to exploring the inequalities in e-cigarette use among Korean adolescents, this study analyzed the crude prevalence of e-cigarette use among adolescents. This study found that the crude rate of e-cigarette use among Korean adolescents showed a fluctuating downward trend. This aligns with previous findings that the strict regulation of e-cigarettes in Korea has been effective^26^. However, the Korean government’s laissez-faire approach to non-nicotine e-cigarettes and limited regulation of e-cigarette online marketing contrast with overall regulatory efforts, hindering the rapid decline in the prevalence of nicotine-containing e-cigarettes among adolescents. In an era of widespread online e-cigarette marketing and rapid development of new tobacco products^27^, policymakers and regulators need to enhance and update regulatory measures to address the prevalence of these products.

This research explored the trend of socioeconomic inequality in e-cigarette use among South Korean adolescents. Our findings indicate a consistently negative Concentration Index from 2011 to 2023, suggesting that e-cigarette use among adolescents is concentrated among those with lower SES. Notably, the Concentration Index demonstrated a significant downward trend over the 13 years, indicating worsening socioeconomic inequalities in e-cigarette use among adolescents. This trend reflects the limitations of Korean e-cigarette regulations, which have been more effective among adolescents with higher SES. This also highlights a significant flaw in global tobacco control policies: the absence of effective measures targeting tobacco use among low-SES populations^28^. Our study provides new evidence on the peer effects influencing adolescent smoking. Peer use of tobacco products not only predicts adolescents’ tobacco use^29^ but also exacerbates socioeconomic inequalities in adolescents’ e-cigarette use among friends and siblings. Adolescents from low SES backgrounds are vulnerable to the temptation of these risky behaviors. Adolescents are highly susceptible to mimicking the behaviors of their peers, especially those from low SES backgrounds^30^. Once an adolescent of low SES starts using e-cigarettes, the entire peer network is at risk, potentially leading to a rapid spread of e-cigarettes within their networks. Adolescents who smoke e-cigarettes are more likely to form friendships, making it harder for them to quit^31^. However, not all peer interactions among adolescents are harmful. A meta-analysis suggests that peer support can improve smoking cessation rates^32^. Therefore, avoiding the negative peer effects on adolescents’ e-cigarette use and stimulating the positive peer effects are important issues, especially for adolescents with low SES. In the future, more research and evidence are needed to validate these findings.

In addition, maternal smoking status significantly contributes to the socioeconomic inequality in e-cigarette use among Korean adolescents. This may be attributed to the fact that Korean adolescents spend a considerable amount of their time at home, where mothers, often the primary caregivers, play a significant role^33^. Moreover, in low SES families, parental smoking likely contributes to the intergenerational transmission of tobacco use. This serious phenomenon indicates ongoing intergenerational health consequences associated with tobacco product use among families of low SES. This underscores the urgent need for policies targeting socioeconomic inequalities in e-cigarette use among adolescents.

### Strengths and limitations

A major strength of this study is that it is the first demonstration of socioeconomic inequalities in e-cigarette use among Korean adolescents and the contribution of smoking by family members and friends to social inequalities. Additionally, this study used parents’ education, adolescents’ academic achievement, and family economic status to construct the adolescents’ SES index, addressing gaps in previous studies that overlooked the importance of adolescents’ circumstances on their SES. Despite its pioneering nature, this study has several limitations. First, this study only considered the role of family and friends’ smoking on socioeconomic inequalities in e-cigarette use, although a multitude of determinants would influence these disparities. Second, this study focuses solely on socioeconomic inequalities in e-cigarette use among adolescents, not on other tobacco products, such as heated e-cigarettes and non-medicated oral nicotine products. Third, as a cross-sectional study, it does not estimate socioeconomic status’s causal or long-term effects on e-cigarette use. Fourth, the computation and decomposition methods of the Concentration Index cannot estimate the causal effects because of the endogeneity problem.

## CONCLUSIONS

Socioeconomic inequality in e-cigarette use among South Korean adolescents has widened from 2011 to 2023. This trend implies that although overall e-cigarette use among Korean adolescents is declining, it is increasingly concentrated among those from lower SES backgrounds. The smoking status of peer groups, including friends and siblings, has the greatest impact on the socioeconomic inequality in e-cigarette use among Korean adolescents, followed by maternal smoking.

## Supplementary Material



## Data Availability

The data that support the findings of this study are available from the Korea Youth Risk Behavior Web-based Survey (KYRBS) at http://yhs.cdc.go.kr.
