# Supplementary material for: Trends in socioeconomic inequality in e-cigarette use among adolescents in South Korea
Source: Tob Induc Dis. 2024 Oct 17;22:10.18332/tid/194099. doi: 10.18332/tid/194099 (PMC11484377; doi:10.18332/tid/194099)
Supplement: Supplementary file 1 [file TID-22-167-s1.pdf]

Table S1 Descriptive statistics 2011-2016 (N=501,133).

Variables	Category	2011	2012	2013	2014	2015	2016
Gender	Boy	50.07%	51.52%	50.60%	50.61%	51.74%	51.59%
	Girl	49.93%	48.48%	49.40%	49.39%	48.26%	48.41%
Age		15.10(1.75)	14.89(1.76)	14.90(1.76)	14.94(1.75)	14.96(1.74)	14.99(1.73)
Family Economic Status	Very Poor	5.16%	5.13%	4.68%	3.78%	3.26%	2.91%
	Poor	17.91%	17.81%	16.30%	14.37%	13.71%	12.70%
	Medium	47.28%	47.02%	47.62%	48.63%	46.97%	47.39%
	Rich	23.34%	23.59%	24.19%	25.44%	26.92%	27.46%
	Very Rich	6.32%	6.45%	7.21%	7.79%	9.13%	9.53%
Father's Education	Junior school	6.03%	5.26%	4.74%	3.69%	3.23%	2.82%
	High school	42.24%	41.88%	41.23%	37.43%	35.86%	35.19%
	University	51.74%	52.86%	54.03%	58.88%	60.90%	61.99%
Mother's Education	Junior school	6.00%	5.11%	4.40%	3.19%	2.72%	2.34%
	High school	54.80%	53.31%	51.53%	47.30%	44.19%	42.54%
	University	39.20%	41.57%	44.08%	49.51%	53.09%	55.12%
Academic Performance	Poor	12.38%	13.30%	12.68%	10.92%	10.89%	10.05%
	Below Average	25.56%	25.53%	25.00%	24.01%	23.51%	23.01%
	Average	26.94%	26.76%	27.82%	27.98%	27.85%	28.42%
	Above Average	24.13%	23.73%	23.54%	24.80%	25.09%	25.26%
	Excellent	10.99%	10.68%	10.96%	12.28%	12.66%	13.26%
SES Index		0.04(0.84)	0.04(0.83)	0.04(0.83)	0.04(0.83)	0.04(0.83)	0.04(0.83)
Father Smoking	Yes				47.43%	43.84%	44.46%
Mother Smoking	Yes				3.27%	3.11%	3.18%
Friends Smoking	Yes				44.91%	40.57%	38.34%
Siblings Smoking	Yes				6.27%	5.57%	5.51%
Grandparents Smoking	Yes				6.96%	6.15%	6.28%

Categorical variables report percentages and numerical variables report means with standard deviations in parentheses.

Table S2 Descriptive statistics 2017-2023 (N=394,145).

Variables	Category	2017	2018	2019	2020	2021	2022	2023
Gender	Boy	50.78%	50.74%	52.08%	51.60%	51.78%	50.91%	50.62%
	Girl	49.22%	49.26%	47.92%	48.40%	48.22%	49.09%	49.38%
Age		15.00(1.75)	15.01(1.77)	14.97(1.78）	15.10(1.75)	15.09(1.74)	15.10(1.74)	15.08(1.73)
Family Economic Status	Very Poor	2.56%	2.40%	2.27%	2.32%	2.03%	1.95%	1.99%
	Poor	11.72%	10.96%	10.54%	10.80%	9.28%	9.27%	10.28%
	Medium	45.90%	46.32%	47.92%	48.04%	49.37%	46.57%	45.35%
	Rich	29.05%	29.45%	28.14%	27.84%	28.49%	30.67%	30.50%
	Very Rich	10.78%	10.87%	11.13%	10.99%	10.84%	11.54%	11.88%
Father's Education	Junior school	2.44%	2.06%	2.10%	2.01%	1.73%	1.64%	1.40%
	High school	32.71%	30.76%	29.78%	28.79%	26.93%	25.07%	24.12%
	University	64.85%	67.18%	68.12%	69.20%	71.34%	73.29%	74.48%
Mother's Education	Junior school	2.04%	1.68%	1.73%	1.44%	1.36%	1.38%	1.10%
	High school	39.29%	35.97%	33.87%	32.99%	30.45%	27.51%	25.99%
	University	58.67%	62.35%	64.41%	65.58%	68.19%	71.11%	72.91%
Academic Performance	Poor	9.83%	9.74%	9.70%	10.07%	9.87%	9.51%	9.30%
	Below Average	22.19%	22.07%	21.94%	23.08%	21.89%	21.95%	23.41%
	Average	28.60%	29.19%	30.08%	30.18%	30.82%	29.86%	29.39%
	Above Average	25.69%	25.57%	24.95%	24.40%	24.51%	25.30%	25.05%
	Excellent	13.69%	13.44%	13.34%	12.26%	12.92%	13.38%	12.85%
SES Index		0.04(0.83)	0.05(0.82)	0.05(0.82)	0.05(0.82)	0.05(0.81)	0.06(0.80)	0.06(0.80)
Father Smoking	Yes		42.92%			41.37%		
Mother Smoking	Yes		3.21%			3.50%		
Friends Smoking	Yes		38.50%			30.72%		
Siblings Smoking	Yes		5.55%			5.39%		
Grandparents Smoking	Yes		6.63%			6.14%		

Categorical variables report percentages and numerical variables report means with standard deviations in parentheses.

Table S3 Decomposition of Concentration Index of e-cigarette in 2014 (N=72,060).

Variables	Elasticity	Concentration index	Contribution	Contribution (%)
	(1)	(2)	(3) = (1) *(2)	(4)
Father Smoking	-0.005	-0.090	0.0004	1.449
Mother Smoking	-0.006	-0.386	0.002	7.438
Friends Smoking	-0.388	-0.058	0.023	77.112
Siblings Smoking	-0.017	-0.238	0.004	13.995
Grandparents Smoking	-0.003	-0.006	0.00002	0.073
Residual				-0.067

Table S4 Decomposition of Concentration Index of e-cigarette in 2015 (N=68,043).

Variables	Elasticity	Concentration index	Contribution	Contribution (%)
	(1)	(2)	(3) = (1) *(2)	(4)
Father Smoking	0.019	-0.095	-0.001	2.485
Mother Smoking	0.015	-0.391	-0.006	7.938
Friends Smoking	0.921	-0.065	-0.060	80.011
Siblings Smoking	0.036	-0.250	-0.009	12.019
Grandparents Smoking	0.008	0.008	0.00007	-0.090
Residual				-2.363

Table S5 Decomposition of Concentration Index of e-cigarette in 2016 (N=62,276).

Variables	Elasticity	Concentration index	Contribution	Contribution (%)
	(1)	(2)	(3) = (1) *(2)	(4)
Father Smoking	0.002	-0.089	-0.0002	-0.721
Mother Smoking	-0.004	-0.411	0.002	6.141
Friends Smoking	-0.331	-0.065	0.022	80.550
Siblings Smoking	-0.014	-0.237	0.003	12.693
Grandparents Smoking	-0.005	0.004	-0.00002	-0.081
Residual				1.419

Table S6 Decomposition of Concentration Index of e-cigarette in 2018 (N=60,040).

Variables	Elasticity	Concentration index	Contribution	Contribution (%)
	(1)	(2)	(3) = (1) *(2)	(4)
Father Smoking	-0.005	-0.104	0.001	1.810
Mother Smoking	-0.005	-0.451	0.002	8.006
Friends Smoking	-0.311	-0.073	0.023	75.847
Siblings Smoking	-0.015	-0.268	0.004	13.618
Grandparents Smoking	-0.00005	-0.016	0.0000009	0.003
Residual				0.716

Table S7 Decomposition of Concentration Index of e-cigarette in 2021 (N=54,848).

Variables	Elasticity	Concentration index	Contribution	Contribution (%)
	(1)	(2)	(3) = (1) *(2)	(4)
Father Smoking	-0.011	-0.116	0.001	4.286
Mother Smoking	-0.004	-0.504	0.002	7.291
Friends Smoking	-0.257	-0.090	0.023	78.727
Siblings Smoking	-0.012	-0.310	0.004	13.065
Grandparents Smoking	0.004	-0.005	-0.00002	-0.061
Residual				-3.307

© 2024 Chen G. et al.
